# Reduced neural suppression at occipital cortex in subthreshold depression

**DOI:** 10.1038/s41398-025-03446-9

**Published:** 2025-07-01

**Authors:** Jinhui Li, Yuheng Tan, Zixin Zheng, Chunliang Feng, Wenjie Fang, Xiaodan Huang, Song Lin, Kwok-Fai So, Lu Huang, Chaoran Ren, Qian Tao

**Affiliations:** 1 Division of Medical Psychology and Behavior Science, School of Medicine, Jinan University, Guangzhou, China; 2 Key Laboratory of Brain, Cognition and Education Sciences, South China Normal University (Ministry of Education), Guangzhou, China; 3 School of Psychology, South China Normal University, Guangzhou, China; 4 Key Laboratory of CNS Regeneration (Ministry of Education), Guangdong Key Laboratory of Non-human Primate Research, GHM Institute of CNS Regeneration, Jinan University, Guangzhou, China; 5 Physiology Department, School of Medicine, Jinan University, Guangzhou, China; 6 Co-innovation Center of Neuroregeneration, Nantong University, Nantong, China; 7 Department of Rehabilitation Medicine, First Affiliated Hospital of Jinan University, Guangzhou, China

**Keywords:** Depression, Human behaviour

## Abstract

Impaired visual perception and biochemical changes in the occipital cortex have been observed in major depression. However, the neural basis underlying these abnormalities remains yet elusive. Importantly, it remains unknown whether these abnormalities are present in the early stage of depression, known as subthreshold depression (SD). Recognized as a precursor of major depression, SD has gained a growing attention in both research and clinical fields. The current study recruit young adults with SD and demographically matched healthy controls (HC). Experiment 1 utilized a series of psychophysical tasks in a large sample (*n* = 95), and Experiment 2 used a functional magnetic resonance imaging (fMRI) approach in a medium sample (*n* = 63). Our results show that the impaired spatial suppression at the behavioural level is accompanied by a significant reduction in neural suppression in the human middle temporal complex (hMT+) and early visual cortex (EVC) within the SD group. Additionally, we found enhanced functional connectivity between hMT+ and the medial prefrontal cortex, anterior cingulate cortex, insula, and postcentral gyrus in the SD group. These findings provide new insights into the psychopathological mechanisms underlying depressive symptoms and highlight the significance of the occipital cortex in the early identification and prevention of depression.

## Introduction

Major depressive disorder (MDD) is a psychiatric illness characterized by mood dysregulation. Previous studies suggested that MDD patients also exhibited abnormalities in visual and attentional functions, such as enhanced motion perception and reduced spatial suppression [1–3], abnormally slow dynamics in visual perception [4–6], reduced retinal contrast gain [6, 7], reduced visual contrast sensitivity [8–10], abnormal pattern glare [11, 12], reduced binocular rivalry [13–15], and reduced attentional control [16]. Noteworthy, there was a significant correlation between reduced spatial suppression and depressive severity [1–3]. In parallel, the occipital cortex has been proposed to play a crucial role in the onset and progression of depression, as well as in the efficacy of antidepressant treatment [17]. For instance, increasing evidence in neuroimaging studies have shown alterations in activity of occipital cortex in MDD while they at rest [18–20] or task state [21–23].

The occipital cortex comprises multiple subregions organized along the visual hierarchy, ranging from early visual cortex (EVC) at the bottom to the human middle temporal complex (hMT+) at the higher hierarchical tiers. Noteworthy, both EVC and hMT+ are well known for their roles in visual motion processing [24, 25], which has been shown to have decreased GABA levels in MDD patients compared to healthy control (HC) individuals [2]. The biochemical changes in occipital GABA among MDD patients further lead alterations in both excitation–inhibition balance [2] and resting-state functional connectivity [3, 26] Importantly, the reduction in occipital GABA in MDD can be normalized following antidepressant treatments [27, 28]. Another resting state fMRI study found reduced neural dynamics in the hMT+ and EVC in MDD patients, and abnormally high functional connectivity between the occipital cortex and other brain regions [29]. Reduced functional connectivity between hMT+ and motor cortex was suggested to mediate psychomotor retardation in MDD [26].

According to the dimensional approach, depressive disorders are viewed as existing along a spectrum of increasing severity [30]. Patients with a longer period of depression have shown less spatial suppression [1], indicating that abnormalities in visual function and the occipital cortex may manifest at an earlier stage of disease, including in cases of subthreshold depression (SD). As a transitional stage between mental health and MDD, SD has increasingly been recognized as a target for intervention to prevent the progression to MDD [31–33]. While depressive symptoms in SD are mild, they are associated with a range of adverse outcomes, including functional impairment, an increased risk for suicide and for developing MDD [34, 35]. Compared to MDD, SD has a higher prevalence and greater health service burden [36]. Despite a growing recognition of the significance of SD over the last decades [36, 37], the visual function status and occipital cortex changes in SD remain largely unknown. To the best of our knowledge, only one study has examined visual functioning in individuals with SD, and the authors found that SD individuals had reduced contrast discrimination sensitivity compared to controls at both initial and four-month follow-up assessments. This suggests the presence of stable abnormalities of visual function in SD individuals [38]. In addition, several neuroimaging studies have indicated abnormalities in the occipital cortex of SD [39, 40].

In the present study, we first aimed to investigate the visual function status in SD individuals through a serious of psychophysical experiments investigating visual perception. Expectedly, SD individuals would demonstrate reduced spatial suppression compared to HC individuals. Then we sought to further examine the neural basis of spatial suppression using a combined psychophysical and functional magnetic resonance imaging (fMRI) approach. Consistent with patterns seen in MDD, we hypothesized that SD individuals would show reduced spatial suppression, as assessed by a motion spatial task. Given the critical roles of the hMT+ and EVC in visual motion perception, we also hypothesized that neural suppression at these occipital regions would be reduced in SD individuals when processing spatial motion information.

## Methods

### Design

**Fig. 1:**
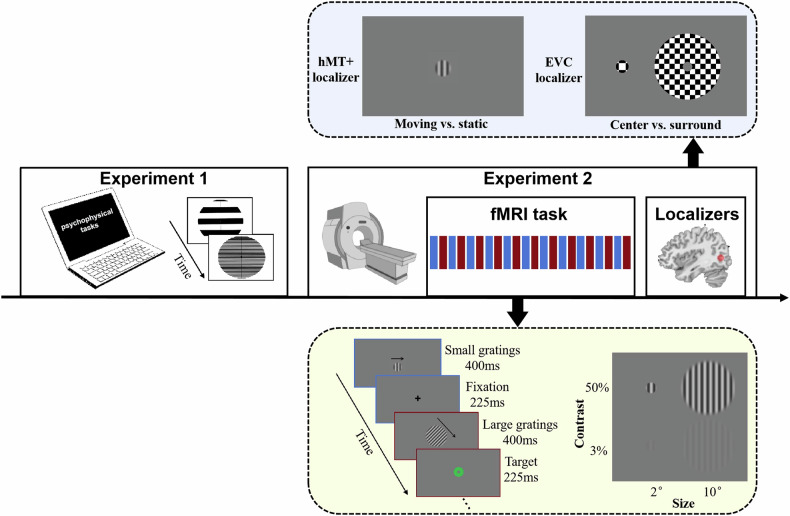
Study design and procedure. Experiment 1 included five psychophysical tasks investigating different aspects of visual perceptual function. Experiment 2 included an fMRI task designed to measure spatial suppression and two localizer scans designed to localize hMT+ and EVC. fMRI functional magnetic resonance imaging, hMT+ human middle temporal complex, EVC early visual cortex.

The study comprised two experiments (Fig. 1) and was conducted between January 2023 and March 2024. Experiment 1 involved a series of psychophysical experiments investigating different aspects of visual function in a large sample of individuals with SD and HC, and it was carried out at Jinan University in Guangzhou, China. Experiment 2 included an fMRI study investigating the neural mechanism underlying visual spatial suppression, and it was conducted at South China Normal University in Guangzhou, China. The authors assert that all procedures contributing to this work comply with the ethical standards of the relevant national and institutional committees on human experimentation and with the Helsinki Declaration of 1975, as revised in 2013. All procedures involving human subjects were approved by the ethics committees of both universities (JUNKY-2023-0044; SCNU-BRR-2023-048). Written informed consents were obtained from all participants, who were reimbursed CNY 60 for their participation in Experiment 1 and CNY 100 for Experiment 2.

### Participants

The sample size was determined on the basis of power calculations using G*Power 3.1 software, assuming a medium to large effect (*d* = 0.7), a statistical power of 85% (α = 0.05), and a minimum required sample size of 30 participates per group. A total of 149 participants were recruited for the study: 44 SD (11 males) participants and 51 HC (14 males) participants in Experiment 1, and 35 SD (20 males) and 38 HC (20 males) in Experiment 2, with 10 SD and 9 HC included in both experiments. Demographics were matched between SD and HC groups across both experiments (Table 1). The screening and recruitment procedures were detailed in our previous studies [41–43]. Briefly, participants were assessed by two online surveys of the Centre for Epidemiologic Studies Depression Scale (CES-D) [44] and the Beck Depression Inventory-II (BDI-II) [45] at the initial screening and again at two-week follow-up. Eligibility was finally confirmed by the 24-item Hamilton Depression Rating Scale (HAMD) [46]. Inclusion criteria were as follows: aged between 18 and 28 years, right-handed, and had normal or corrected-to-normal visual acuity without visual impairment. Participants were excluded from the study if they had: (a) any other mental disorders as defined by the Diagnostic and Statistical Manual of Mental Disorders, 5th edition DSM-5 [47]; (b) suicidal tendencies; (c) ophthalmic diseases such as cataracts, glaucoma, retinitis pigmentosa, or diseases affecting the retina; (d) received any form of depression intervention in the past year; (e) a diagnosis of serious diseases requiring interventions, such as neurological disease or head injury; (f) or any magnetic resonance imaging (MRI) contra-indications.

**Table 1:** Demographic and clinical characteristics of participants.

	Experiment 1			Experiment 2		
Characteristics	SD (*n* = 44)	HC (*n* = 51)	*p*	SD (*n* = 33)	HC (*n* = 30)	*p*
Age, mean (SD)	21.73 (2.57)	21.59 (2.80)	0.803	20.52 (2.09)	21.47 (2.39)	0.097
Gander, No (%)						
Male	11 (25.00%)	14 (27.45%)	0.819	13 (39.39%)	15 (50.00%)	0.397
Female	33 (75.00%)	37 (72.55%)		20 (60.60%)	15 (50.00%)	
Education years, mean (SD)	15.80 (2.41)	15.33 (2.26)	0.338	14.66 (1.92)	15.24 (2.02)	0.130
Major, No (%)						
Science	5 (11.36%)	4 (7.84%)	0.264	4 (12.12%)	4 (13.33%)	0.502
Liberal art	10 (22.72%)	6 (11.76%)		10 (30.30%)	13 (43.33%)	
Medicine	29 (65.90%)	41 (80.40%)		19 (57.58%)	13 (43.33%)	
Depressive score, mean (SD)						
CES-D	25.95 (7.20)	7.02 (4.13)	<0.001	25.33 (8.67)	8.82 (3.73)	<0.001
BDI-II	21.07 (6.72)	4.31 (3.46)	<0.001	21.42 (5.16)	6.42 (4.14)	<0.001
HAMD	11.11 (2.72)	1.63 (1.59)	<0.001	13.20 (2.29)	4.12 (2.20)	<0.001
Visual acuity, mean (SD)						
Left eye	4.51 (0.46)	4.46 (0.37)	0.635	4.52 (0.34)	4.45 (0.42)	0.463
Right eye	4.47 (0.44)	4.40 (0.35)	0.379	4.47 (0.35)	4.38 (0.36)	0.301
Corrected vision, mean (SD)						
Left eye	5.06 (0.05)	5.06 (0.05)	0.592	5.06 (0.05)	5.08 (0.43)	0.174
Right eye	5.06 (0.05)	5.05 (0.08)	0.572	5.05 (0.07)	5.06 (0.07)	0.292

*CES-D* centre for epidemiologic studies depression scale, *BDI-II* beck depression inventory-II, *HAMD* hamilton depression rating scale.

### Experiment 1

To account for factors that might affect visual performance, visual acuity was assessed in all participants using the GB/T 11533-2011 standard for logarithmic visual acuity charts. To investigate different aspects of visual function, participants underwent a series of psychophysical experiments, with the order of experiments was counterbalanced across the participants. Visual motion and spatial suppression were tested by a spatial suppression paradigm based on previous studies [24, 48]. The strength of spatial suppression was quantified using the spatial suppression index (SI), defined as the difference in log_10_ thresholds for large versus small stimuli. A higher SI indicates stronger spatial suppression.

$${\rm{SI}}={\log }_{10}({\rm{large\; threshold}})-{\log }_{10}({\rm{small\; threshold}})$$

In addition, visual contrast, visual competition, spatial perception, and visual stress were assessed by relevant tasks. These visual assessments were conducted on a 14-inch monitor Lenovo laptop monitor (1920 × 1080 pixels; 60 Hz refreshment rate). Visual stimuli were created and presented using MATLAB (MathWorks, Natick, MA), E-prime 3.0 (Psychology Software Tools, Pittsburgh, PA) and Psych Toolbox (Open Science Tools, Nottingham, England). All psychophysical experiments took place in a quiet room with dim light, and participants were allowed a 10 min adaptation period to the lighting condition before testing. Participants were seated comfortably at an approximate distance of 60 cm from the screen, using a chin rest to ensure stable head positioning. Additional details are provided in the Supplementary Methods.

### Experiment 2

We replicated the spatial suppression paradigm with an fMRI design using a small sample in Experiment 2 [24, 49]. Stimuli were presented within the central 2° of the screens. The experiment utilized a block design using Psychopy 2.3 (Open Science Tools, Nottingham, England). In a high contrast condition (50%), we displayed small (2° diameter) and large (10° diameter) drifting gratings (drift rate = 4 cycles/s, spatial frequency = 1 cycle/°, Gaussian envelope standard division = 0.25°) at the centre of the screen, organized into alternating 10 s blocks. These visual stimuli parameters were derived from the previous studies [2, 24, 48], which have been shown to be effective in triggering spatial suppression effects and are suitable for investigating the neural mechanisms of visual motion perception. Each block comprised sixteen 625 ms trials, with each trial featuring of a 400 ms grating presentation followed by a 225 ms inter-stimulus interval (ISI). The gratings drifted in one of eight possible directions, with the order randomized and counterbalanced. A single fMRI run lasted 4.17 min, encompassing a total of 25 blocks (13 small gratings and 12 large gratings). A low contrast task (3%) followed a similar structure, differing only in the contrast level. Participants completed three fMRI runs at both low and high contrast levels. To ensure attention, participants were required to perform a matching task with consideration of both colour and shape by responding to a target among distractors. The shapes, each with a diameter of 0.5°, were presented at the center for 66 ms every 1333 ms, overlapping with the drifting gratings. Behavioural data from the colour/shape matching task with a correct rate below 70% was excluded from the analysis.

To identify the regions of interest (ROI), two functional localizer scans were used. The hMT+ localizer scan [24, 48] consisted of alternating 10 s blocks of drifting and static gratings (2° diameter, 15% contrast), comprising a total of 25 blocks with 13 static and 12 drifting blocks. Each grating was displayed for 400 ms, followed by a 225 ms ISI. The EVC localizer scan [24, 48] presented checkerboard stimuli in the centre and surrounding regions in an alternating order across sixteen 10 s blocks. The centre stimuli had a diameter of 2°, while the surrounding stimuli had inner and outer diameters of 2° and 12°, respectively.

All imaging data were collected on a 3 T Siemens Prismafit scanner with a 64-channel phased-array head/neck receiver coil. The fMRI data were obtained using a single-shot simultaneous multi-slice or multiband gradient-echo EPI sequence with the following parameters: repetition time (TR) = 2000 ms, echo time (TE) = 30 ms, flip angle = 90°, slice acceleration factor = 2, field of view (FOV) = 224 mm × 224 mm, voxel size = (2 mm)^3^, data matrix = 112 × 112, slice thickness = 2 mm without inter-slice gap, anterior-to-posterior phase encoding direction, and 60 interleaved slices covering the whole brain. In addition, high-resolution brain structural images were acquired using a T1-weighted 3DMP-RAGE sequence with the following parameters: TR = 2530 ms, TE = 1.94 ms, flip angle = 7°, slice thickness = 1.0 mm, FOV = 256 mm × 224 mm, voxel size = 0.5 mm × 0.5 mm × 1.0 mm, data matrix = 256 × 224, and 208 sagittal slices covering the whole brain.

### Data analysis and statistics

Functional images were preprocessed using SPM12 (https://www.fil.ion.ucl.ac.uk/spm/software/spm12/). The steps included: slice-timing correction, head motion correction, co-registration, segmentation, normalization, and spatial smoothing with an 8-mm full width at half maximum (FWHM) Gaussian kernel. We then performed a general linear model (GLM) to analyze the fMRI data. In the first-level analysis, the time series for the large gratings were modeled as a convolution of a boxcar with a canonical hemodynamic response function (HRF). The small gratings served as the baseline. In addition, six motion parameters were included in the GLM to reduce the effects of head motion. A high-pass filter with a cutoff of 1/128 Hz was applied to remove low frequency drifts.

For ROI delineation, we selected peak voxels from the intersection of the motion-selective response in the first localizer task and the hMT+ atlas to create a 7-mm sphere hMT+ mask. Similarly, peak voxels from the intersection of the centre-selective response in the second localizer task and the EVC atlas to create a 7-mm sphere EVC mask. The ROI positions were verified by visualizing each individual’s inflated brain model in relation to anatomical features of hMT+ and EVC. Examples of the defined ROIs in hMT+ and EVC from six participants were demonstrated in Fig. S1. With a focus on changes in fMRI signal when stimulus size transitioned from small to large, we extracted β coefficients from these ROIs for statistical analysis. In both high and low contrast conditions, we defined the time at which the stimulus size change as 0 s (event-related time = 0 s). The ROI time series data were divided into epochs ranging from 4 s before the stimulus became smaller to 12 s after the stimulus became larger. The baseline fMRI response was calculated by averaging the signals from 0–4 s prior to the size change across all epochs. We then performed baseline correction by subtracting each time series data from the mean of baseline epochs. The magnitude of the fMRI response was derived from the average signal from 8–12 s after the stimulus size increased [24, 48].

We conducted psychophysiological interaction (PPI) analysis [50] to examine the functional connectivity underlying the visual motion task. Specifically, fMRI signal time series were extracted from the ROIs to serve as seeding signals. These seeding signals were then deconvolved with the canonical HRF to produce an estimate of potential neuronal activity. Subsequently, we computed interactions between the estimated neuronal time series and each starting point vector representing the regressors. Finally, these interaction terms were reconvolved with the HRF and entered a new GLM along with the starting vectors (the psychological terms) for each event. The group-level analysis of the PPI data followed a similar approach to that of the brain activation data, with the *β* values derived from the PPI regressors. The PPI analysis was conducted with a threshold of voxel-level *P* < 0.001 and a cluster level family-wise error (FWE) correction (*P*_FWE_ < 0.05).

For the contrast sensitivity task, we employed linear mixed-effects modeling (LMM) to examine differences between the SD and HC groups, both group and spatial frequency were added as fixed effects, with participant as a random intercept and age as a covariate. For the spatial frequency task, an LMM was used to investigate between-group differences, treating group as the between-subjects variable, spatial frequency as the within-subjects variable, and age as a covariate. For the binocular rivalry and pattern glare tasks, we used two sample *t*-tests and Mann-Whitney *U*-test to investigate between-group differences, respectively. Statistical analyses of the behavioural data were performed using the SPSS (version 19.0) and R (version 4.4.1). Levene’s test and Bartlett’s test were used to confirm the similarity of variances between the groups.

## Results

### Behavioural results

**Fig. 2:**
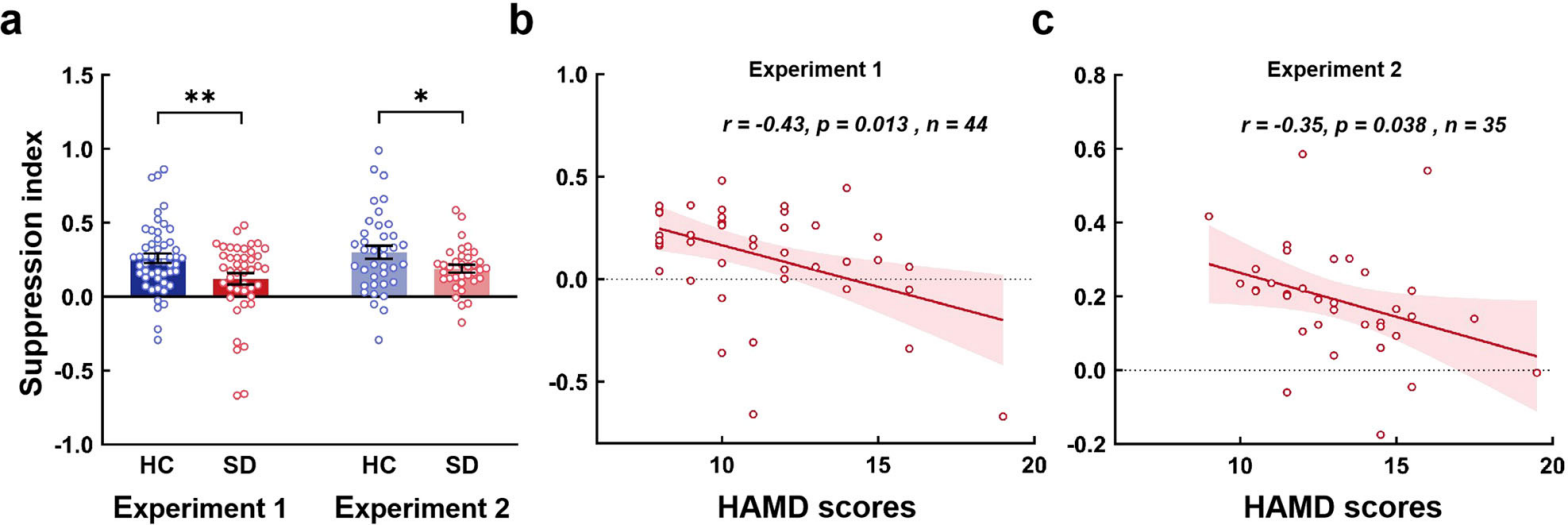
Behavioural results for the spatial suppression task. **a** The results for a large sample in Experiment 1 (*n* = 95) and a small sample in Experiment 2 (*n* = 73). For Experiment 1, the mean motion SI was significantly lower in SD group (0.12 ± 0.04) compared to HC group (0.26 ± 0.03) (*p* = 0.008). For Experiment 2, the mean motion SI was significantly lower in SD group (0.19 ± 0.03) compared to HC group (0.30 ± 0.04) (*p* = 0.031). **b** For the SD participants in Experiment 1, there is a significant negative correlation between the motion SI and HAMD scores (*r* = −0.43, *p* = 0.013). **c** For the SD participants in Experiment 2, there is a significant negative correlation between the motion SI and HAMD scores (*r* = −0.35, *p* = 0.038). SI suppression index, HAMD hamilton depression rating scale. *p < 0.05, **p < 0.01. Error bars are S.E.M.

There were no significant between-group differences in the duration thresholds in either Experiment 1 (large sample) or Experiment 2 (small sample). Importantly, the SI was significantly smaller in the SD group (*M* ± *SD* = 0.12 ± 0.25 for Experiment 1; *M* ± *SD* = 0.19 ± 0.15 for Experiment 2) than in the HC group (*M* ± *SD* = 0.26 ± 0.23 for Experiment 1; *M* ± *SD* = 0.30 ± 0.27 for Experiment 2; *t*_91_ = 2.72, *p* = 0.008 for Experiment 1; *t*_71_ = 2.20, *p* = 0.031 for Experiment 2) (Fig. 2a). Moreover, the SI was negatively correlated with HAMD score in the SD group for both experiments (*r* = −0.43, *p* = 0.013 for Experiment 1; *r* = −0.35, *p* = 0.038 for Experiment 2) (Fig. 2b, c). In the contrast sensitivity task, we found a nonsignificant main effect of group (*t*_184_ = 0.14, *p* = 0.889) and a nonsignificant interaction between group and spatial frequency (*t*_93_ = 1.66, *p* = 0.100). However, there was a significant main effect of spatial frequency (*t*_93_ = 2.64, *p* = 0.010). Age was a nonsignificant covariate (*t*_93_ = −1.20, *p* = 0.230). In the binocular rivalry task, we did not find a significant difference in rivalry rates between the SD (*M* ± *SD* = 0.66 ± 0.21) and HC groups (*M* ± *SD* = 0.67 ± 0.21; *t*_93_ = 0.27, *p* = 0.790). In the spatial frequency task, the results revealed a significant main effect of spatial frequency (medium spatial frequency: *t*_186_ = 5.28, *p* < 0.001; high spatial frequency: *t*_186_ = −6.44, *p* < 0.001). Age was a nonsignificant covariate (*t*_92_ = 1.85, *p* = 0.068). There was no significant main effect of group (*t*_195_ = −0.45, *p* = 0.653) or interaction effect between group and spatial frequency (medium spatial frequency: *t*_186_ = 0.48, *p* = 0.631; high spatial frequency: *t*_186_ = −0.13, *p* = 0.897). In the pattern glare task, we found significant between-group differences for medium (z = −2.87, *p* = 0.004) and high spatial frequency gratings (z = −3.43, *p* < 0.001) (Fig. S2).

### fMRI results

**Fig. 3:**
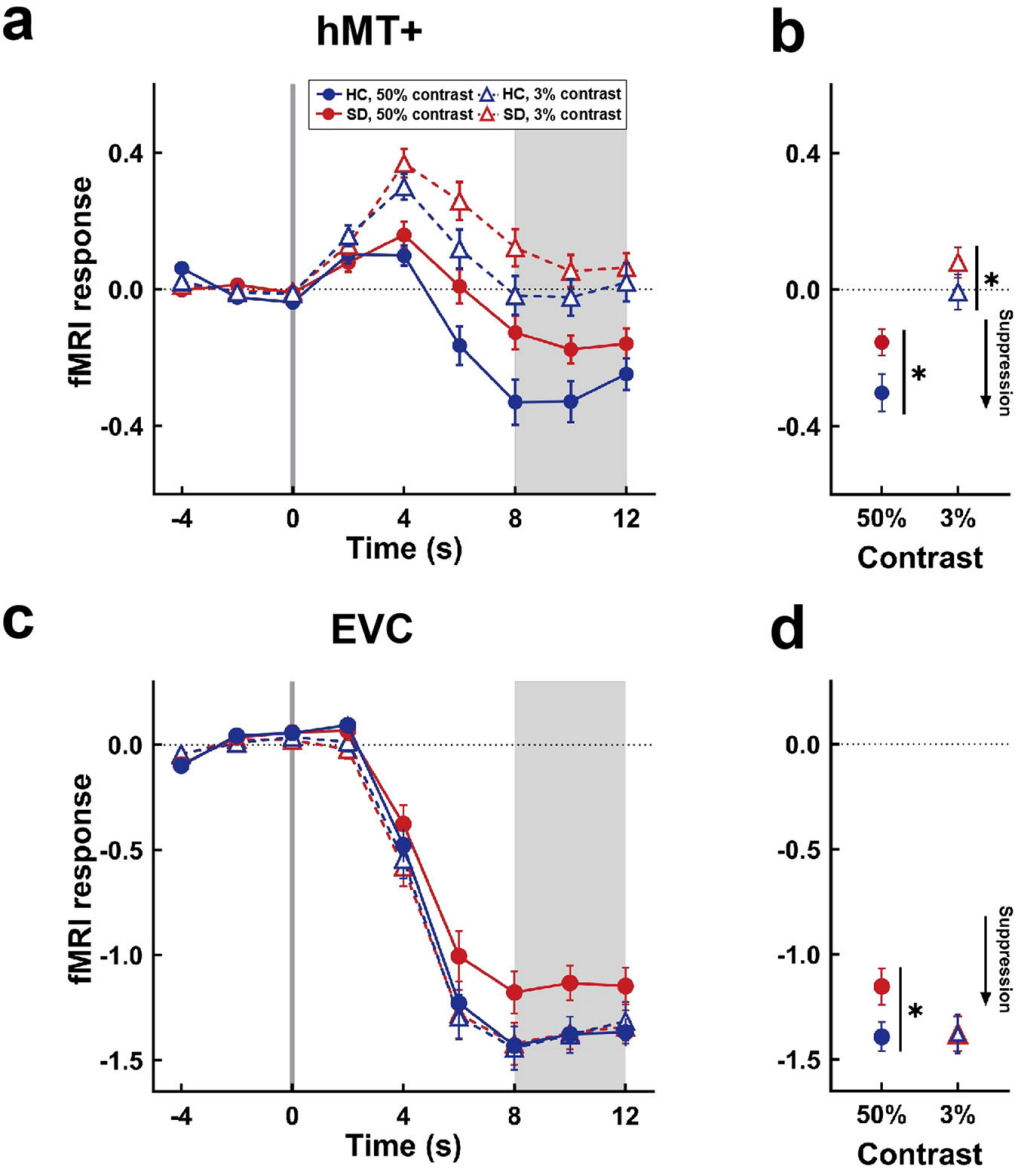
fMRI results for spatial suppression task. **a** fMRI signal at hMT+ in response to an increase in stimulus size (small to large), at time = 0 s; **b** Average fMRI signal at hMT+ for SD and HC groups; **c** fMRI signal at EVC in response to an increase in stimulus size (small to large), at time = 0 s; **d** Average fMRI signal at EVC for SD and HC groups. fMRI functional magnetic resonance imaging; hMT+ human middle temporal complex, EVC early visual cortex, SD subthreshold depression, HC healthy control. *p < 0.05. Dots show group means; error bars are S.E.M.

**Fig. 4:**
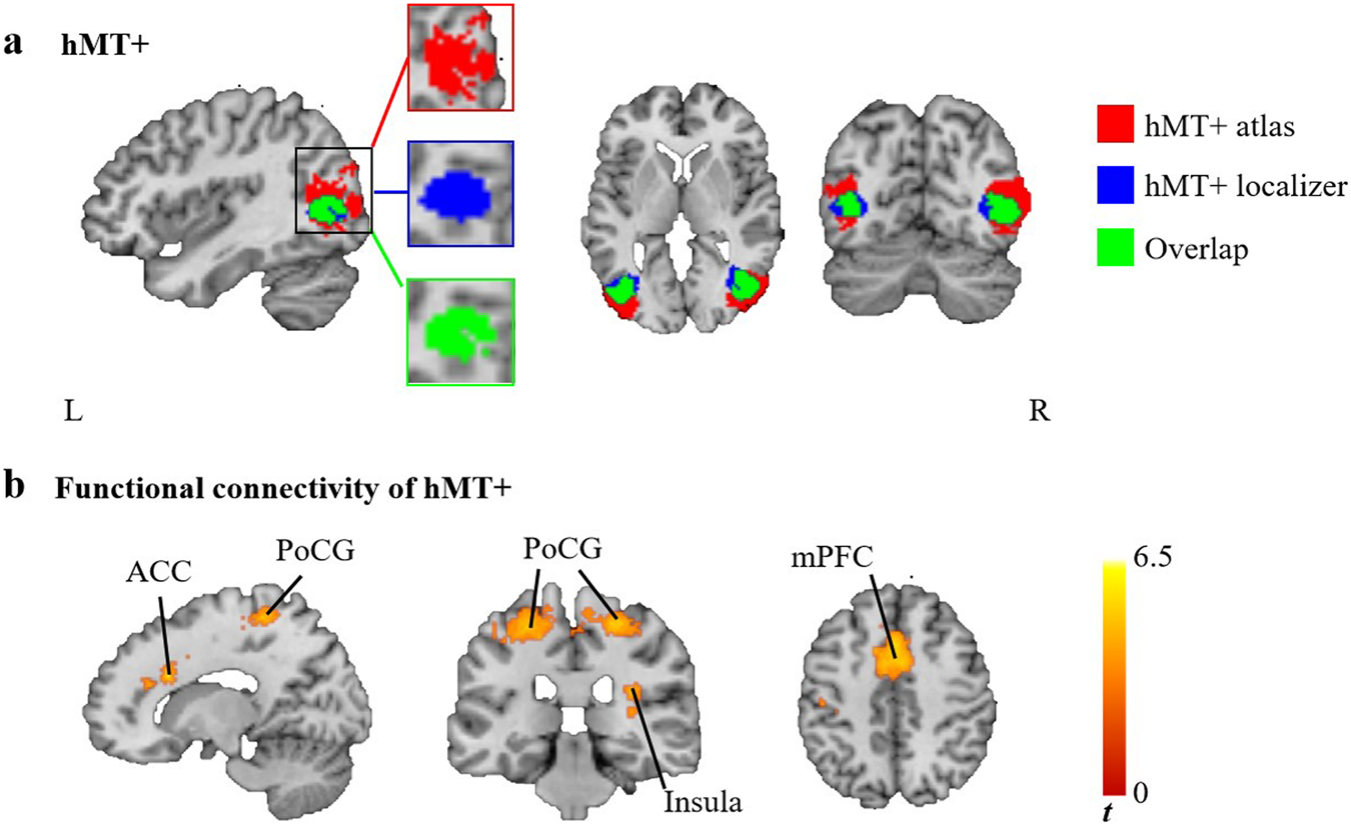
ROI and PPI results. **a** hMT+ in this study. Red, hMT+ defined by the atlas. Blue, hMT+ from functional localizer task. Green, the overlap between the hMT+ defined by the atlas and the hMT+ derived from the functional localizer task; **b** Functional connectivity analysis with hMT+ as a seed region revealed enhanced functional connectivity in the SD group compared to the HC group. ROI region of interest, PPI psychophysiological interaction; hMT+ human middle temporal complex, SD subthreshold depression, HC healthy control, ACC anterior cingulate cortex, PoCG postcentral gyrus, mPFC medial prefrontal cortex, L left, R right.

Due to low accuracy (<70%) in the fMRI task (*n* = 4), large head motion (translation > 2 mm in any of the planes, or rotation > 2° in any of the axes, *n* = 4), and participant fatigue during scanning (*n* = 2), fMRI analyses were conducted on 63 participants (SD = 33, HC = 30). First, we examined the fMRI response within the hMT+. The fMRI responses to large stimuli in hMT+ were significantly lower than baseline in the high contrast condition (*M* ± *SD* = −0.22 ± 0.27; paired *t* test, *t*_62_ = 6.59, *p* < 0.001), but this effect was not significant in the low contrast condition (*M* ± *SD* = 0.04 ± 0.27; paired *t* test*, t*_62_ = 1.19, *p* = 0.272). There were significant main effects of group (*F*_(1__,__61)_ = 4.08, *p* = 0.048) and contrast (*F*_(1__,__61)_ = 61.65, *p* < 0.001), with significantly weaker hMT+ suppression in the SD group than in the HC group and significantly stronger suppression for the high contrast than low contrast condition. But the interaction effect of group and contrast was not significant (*F*_(1__,__61)_ = 0.83, *p* = 0.366) (Fig. 3a, b). Next, we examined the fMRI response within the EVC. We found that fMRI responses in the EVC were significantly suppressed below baseline in both high (*M* ± *SD* = −1.27 ± 0.46; paired *t* test, *t*_62_ = 21.85, *p* < 0.001) and low contrast conditions (*M* ± *SD* = −1.38 ± 0.49; paired *t* test, *t*_62_ = 22.54, *p* < 0.001). The interaction between group and contrast was significant (*F*_(1__,__61)_ = 4.93, *p* = 0.030), but the main effects of group (*F*_(1__,__61)_ = 1.29, *p* = 0.260) or contrast (*F*_(1__,__61)_ = 3.92, *p* = 0.052) were not significant (Fig. 3c, d). Further post hoc analysis revealed that EVC suppression was stronger in the SD than in the HC group for the high contrast condition (*F*_(1__,__61)_ = 4.48, *p* = 0.038), with no significant between-group difference for the low contrast condition (*F*_(1__,__61)_ = 0.00, *p* = 0.995). Compared to the HC group, the SD group demonstrated enhanced functional connectivity between the hMT+ and bilateral postcentral gyrus (PoCG), right anterior cingulate gyrus (ACC), medial prefrontal cortex (mPFC), and insula (Fig. 4 and Table S1). No significant functional connectivity results were found when the EVC was used as the seed region.

The significant gender imbalance observed among SD participants in Experiment 2 raised concerns regarding the generalizability of the findings. To address this concern, we analysed the impact of gender on spatial suppression indices in Experiment 2. For the SD group, there was no significant difference in the spatial suppression index between male and female participants (*t*_33_ = 0.16, *p* = 0.874). Furthermore, we conducted repeated measures ANOVA on the neural responses within the hMT+ and EVC in the SD group, with contrast as the within-subject factor and gender as the between-subject factor. With regards to hMT+ activity, the main effect of contrast was significant (*F*_(1,31)_ = 27.81, *p* < 0.001), the main effect of gender was not significant (*F*_(1,31)_ = 0.50, *p* = 0.485), and the interaction effect was not significant (*F*_(1,31)_ = 0.04, *p* = 0.850). Similarly, for the EVC activity, the main effect of contrast was significant (*F*_(1,31)_ = 9.08, *p* = 0.005), the main effect of gender was not significant (*F*_(1,31)_ = 1.96, *p* = 0.171), and the interaction effect was not significant (*F*_(1,31)_ = 0.00, *p* = 0.974). Taken together, there were no significant differences across gender at both behavioural and neural responses among SD individuals in Experiment 2.

## Discussion

We investigated visual perceptual functions in a large SD sample and neuronal activity in a small SD group using a range of psychophysical tasks and a task-based fMRI approach, respectively. The investigations were replicated and compared with a group of demographically matched HC individuals. The main findings are: (i) SD exhibited reduced spatial suppression at the behavioural level compared to HC individuals in both the large and small samples, with significant correlations between spatial suppression and depressive scores; (ii) reduced neural suppression was observed in both hMT+ and EVC for the SD compared to HC group at high contrast condition; (iii) the SD group showed enhanced functional connectivity between hMT+ and mPFC, ACC, insula, and PoCG compared to HC group; (iv) a significant higher level of visual stress was observed in SD than HC group. Overall, our findings demonstrate reduced spatial suppression at both behavioural and neuronal levels in SD individuals. These results provide new evidence suggesting that impairments in visual perceptual function and occipital cortex may be present in an early stage of depression.

### Weakened spatial suppression in SD

The spatial suppression task is a widely used paradigm that has been applied in people with various psychiatric disorders, including autism [48, 51], schizophrenia [52] and MDD [1–3]. We utilized this task to investigate differences in visual motion perception between SD and HC individuals. As expected, we observed a reduced spatial suppression effect in the SD group, along with a significant correlation between spatial suppression and depressive scores. These findings are consistent with previous studies in MDD [2]. The reduced spatial suppression in SD individuals may be linked to alterations in the top-down modulation of spatial attention. Even at an early stage of MDD, SD individuals exhibit attenuation in the control of top-down spatial attention [53, 54]. When the control of top-down spatial attention is weakened, individuals may focus their attention on a smaller spatial area, producing a larger perceptual blind area and reducing the suppression effect for larger stimuli [48]. However, we found no significant between-group difference in duration thresholds for both large and small gratings. Previous studies have reported inconsistent results regarding duration thresholds in MDD. Some studies indicated higher thresholds in MDD compared to HC [1], while others found comparable duration thresholds between the two groups [2, 3]. The milder depressive symptoms in SD individuals may not have substantially impacted motion perception, whereas more severe depressive symptoms in MDD may significantly affect performance on the spatial suppression task.

### Neural mechanisms underlying weakened spatial suppression in SD

The fMRI results revealed reduced neural suppression in hMT+ for high contrast stimuli, consistent with the spatial suppression effect observed in the behavioural psychophysical experiments in MDD [1–3]. Abnormalities in hMT+ functioning have been reported in MDD patients when processing visuospatial information [1, 2], which may lead to difficulties in effectively suppressing irrelevant stimuli, thereby affecting visual perceptual functions. Our findings in conjunction with previous work, suggest that impaired spatial suppression in hMT+ may serve as a crucial biomarker of altered visual perception, even at an early stage of depression.

We also observed reduced fMRI suppression in the EVC for high contrast stimuli in SD individuals. This finding aligns well with previous studies that reported suppression in the EVC in healthy individuals [24] as well as in animal models [55]. Geniculocortical feedforward and extrastriate feedback connections between EVC and higher order occipital cortex are critical for spatial perception [56]. Furthermore, top-down spatial attentional modulation affects different regions of the visual cortex. Generally, higher-order regions such as hMT+ have larger top-down modulatory effects, while the EVC exhibits smaller top-down modulatory effects [57]. It seems that certain aspects of motion perception in hMT+ may originate from early visual processing in EVC [24].

### Altered functional connectivity in SD

The PPI analysis revealed enhanced functional connectivity between hMT+ and the mPFC in SD group. Abnormalities in the functioning of the mPFC, a crucial region for emotion regulation, have been identified in several studies of MDD [58–60]. For instance, an fMRI study using a reward task showed reduce activity in the mPFC in SD group compared to HC group [61]. Reduced functional connectivity between mPFC and hMT+ was reported in MDD patients in a resting-state fMRI study [3]. Furthermore, transcranial direct current stimulation (tDCS) applied to the dorsolateral PFC has been shown to reverse selective impairments in visual processing speed in major depression [62]. The enhanced mPFC-hMT+ connectivity during visual motion processing may reflect a compensatory mechanism for visual perceptual impairments in SD, who may rely on additional cognitive resources when processing visual motor information [63–65].

Besides the mPFC, we also found enhanced functional connectivity between hMT+ and the ACC, insula, and PoCG in the SD group compared to the HC group. The ACC and insula are both functionally and structurally connected [66, 67], together constituting the salience network [68]. A recent study employing precision functional mapping approach on longitudinal neuroimaging data revealed an expansion of the salience network in depression [69]. Importantly, the authors further revealed a correlation between connectivity changes within the salience circuit and depressive severity over time, suggesting that changes in the salience network may emerge early and predict the onset of depression [69]. Moreover, altered functional connectivity between the frontoparietal network and the occipital cortex has been identified in MDD patients while performing a non-emotion visual task [23]. Our findings of increased functional connectivity between hMT+ and ACC and insula suggest that SD individuals may need to allocate additional attentional resources during visual tasks to compensate for potential deficits in attentional control.

### Visual perceptual functions in SD

Accumulating evidence indicates that visual perception is altered in MDD [5, 6], with impaired visual perceptual functions including visual contrast discrimination [6], visual attention [16], and functional vision [70]. Recovery from depression following interventions has been associated with the normalization of these visual functions [7]. It has been speculated that these altered visual perceptions contribute to core symptoms in depression, such as loss of interest or anhedonia [71, 72]. However, we only found deficits in SD group regarding visual stress, as assessed by the pattern glare task. No significant differences were found in other visual perceptual functions. The pattern glare test is widely used in a variety of neurological conditions, including autism [73], migraine [74, 75], multiple sclerosis [76], stroke [77], and MDD [11]. Our findings on the pattern glare test align well with symptoms reported in depressive individuals, such as headache and photophobia [75, 78].

### Clinical implications

Several studies found that changes in occipital cortex were correlated with improvements following therapeutic interventions in MDD patients, such as primary visual cortex correlated with antidepressant drugs and cognitive behavioural therapy [79, 80]. Neuromodulation technique, such as repetitive transcranial magnetic stimulation (rTMS) and tDCS, is considered a safe and effective treatment for MDD patients [81]. For instance, a study applied rTMS to visual cortex for five days, and the intervention group demonstrated greater effect on reducing depressive symptoms than the sham group both in the treatment period and 4-week follow-up [82]. In addition, changes in task-related activity of visual cortex were correlated with symptomatic reduction, suggesting therapeutic potential and biomarkers of occipital cortex in MDD [82]. By employing a visual motion task and focusing on EVC and hMT+ occipital regions, the current results found reduced spatial suppression at both behavioural and neuronal levels in SD individuals. This highlights the potential value of visual motion task in assessing and identifying depressive individuals at an early disease stage, and the potential therapeutic value of EVC/hMT+ in treating depressive symptoms.

## Conclusion

The present findings reveal reduced spatial suppression at both behavioural and neuronal levels in the SD group compared to HC group. The EVC and hMT+ are key regions underlying the reduced spatial suppression in SD individuals. Furthermore, the SD group demonstrated enhanced functional connectivity between the hMT+ and the mPFC, ACC, insula, and PoCG. Taken together, these results indicate that abnormalities in visual perception and functioning of the occipital region may already be present at the SD stage. This has important implications for the early identification and intervention of depression individuals.

## Supplementary information

Supplement Information

Table S1

## Data Availability

Preprocessed fMRI data, ROI masks, and all behavioural data for the psychophysical tasks are available from the corresponding author upon reasonable request. Behavioural data analysis code and fMRI data analysis code are available on OSF platform via https://osf.io/68trj/.

## Code availability

Preprocessed fMRI data, ROI masks, and all behavioural data for the psychophysical tasks are available from the corresponding author upon reasonable request. Behavioural data analysis code and fMRI data analysis code are available on OSF platform via https://osf.io/68trj/.
