# Supplementary material for: Reduced neural suppression at occipital cortex in subthreshold depression
Source: Transl Psychiatry. 2025 Jul 1;15:220. doi: 10.1038/s41398-025-03446-9 (PMC12216281; doi:10.1038/s41398-025-03446-9)
Supplement: Supplementary file 1 — Supplement Information [file 41398_2025_3446_MOESM1_ESM.docx]

**Supplementary Information**

**Reduced neural suppression at occipital cortex in subthreshold depression**

**Supplementary Methods**

*Psychophysical experiments*

As for the spatial suppression experiment, the stimuli consisted of vertically oriented drifting sinusoidal gratings of small (diameter of 2°) or large (diameter of 10°) size, presented in the center of screen. The gratings had a contrast of 50%, spatial frequency of 1 cycle per degree (c/d), and a drifting speed of 4° per second. The gratings drifted either leftward or rightward, and participants were instructed to decide the direction of motion by pressing the left or right arrow key. Each trial commenced with a crosshair (250 ms) at the center of the screen, followed by the drifting gratings and a response period. To achieve a certain level of performance, the duration for the gratings was adaptively adjusted using a 3-down/1-up staircase procedure based on the participants’ responses. Each session included 160 trials for both small and large stimuli, beginning with several practice trials. For each participant, the correct rate for different stimulus durations was calculated separately. These values were fitted to a cumulative Gaussian function, and the duration thresholds for each stimulus size were estimated at the 75% correct point on the psychometric function [1].

Contrast sensitivity (CS) is an important visual function, and it has been defined as the sensitivity to the relative difference in luminance of an object from its background [2]. We used a contrast sensitivity task to assess visual contrast. The horizontally sinusoidal modulated gratings with two spatial frequencies of 0.5 cycle per degree (c/d) or 4 c/d were presented on the left or right side of the computer screen in a random order. Participants were required to indicate the position of the grating stimulus by pressing a left or right arrow key. Each trial began with a black cross fixation mark (250 ms) at the center of the screen, followed by a horizontal sine-wave grating (500 ms), and then a response period (no time limit). The contrast of each grating pattern was initially set to 50%. Using a 3-down/1-up staircase procedure, the contrast was adjusted based on the participant's responses. The contrast decreased by 0.3 log units following three consecutive correct responses, and increased by the same amount following a single incorrect response. To maintain a certain level of performance, the contrast size was changed to 0.15 log-unit steps following two consecutive incorrect responses [3]. The experiment consisted a practice block and an experiment block, with 50 trials for each block. In the staircase procedure, the mean of the 10 reverse contrast values of the contrast variation determines the contrast threshold for each spatial frequency. The CS was converted to log_10_ CS for analyses, and a larger log_10_ CS indicates better performance.

The binocular rivalry task was used to assess visual competition [4]. The stimuli were composite images (luminance: 135 cd/m^2^; contrast: 90%) of a red concentric ring (8 c/d) and a green radial grating (8 polar cycles), which were presented at a dark gray background (luminance: 30 cd/m^2^). The stimuli were viewed through a red–green anaglyph glass, with a red filter over the right eye and a green filter over the left eye. Participants were instructed to maintain fixation at the center of each composite image, and to respond by pressing the left and right arrow key when the image of the green radial grating and the red circular grating was dominantly perceived, respectively. Each trial lasted 120 s and there were three test trials separated by a break lasting 2 minutes. The rivalry rate (Hz) was calculated as the number of perceptual alterations divided by the total viewing time (seconds).

The spatial frequency discrimination task was used to evaluate spatial perception [5, 6]. The experiment consisted of five blocks, including two practice blocks and three experiment blocks. The practice blocks were used to familiarize participants with the stimuli and procedure, and the three experiment blocks evaluated visual discrimination performance at low (0.5 c/d), medium (4.7 c/d), and high (8.3 c/d) spatial frequency. Each experiment block contained 160 trials (80 with identical and 80 with different spatial frequencies). Each trial began with a black cross fixation (500 ms) at the center of the screen, followed by a grating pair (1500 ms), and then a response period with no time limit. The grating pair consisted of two vertical gratings (luminance: 135cd/m^2^; contrast: 80%) separated by a black border, and the two gratings were randomly changed at spatial frequency and phase. Participants were required to determine whether the grating pair was matched or not on spatial frequency. The performance was indexed by the number of correct responses.

The pattern glare test was used to assess visual perceptual distortions and visual stress when viewing repetitive striped patterns [7, 8]. The stimuli consisted of three patterns that differed at their spatial frequency: low (0.3 c/d), medium (2.3 c/d), and high (9.4 c/d) spatial frequency. The stimuli were presented in the order from low to high spatial frequency. The first pattern of low spatial frequency was to ensure the participants provided accurate responses. The second pattern of medium spatial frequency was to generate the greatest visual distortion in the participants. The third pattern of high spatial frequency was expected to generate fewer symptoms than the second pattern. The participants were instructed to concentrate on a fixation dot in the center of the pattern for 5 seconds, and then they were required to report whether the following symptoms were experienced or not: colors (red, green, blue, and yellow), bending/blurring/shimmering of lines, shadowy shapes among the lines, flickering, fading, pain, discomfort, nausea, dizziness, unease. The number of symptoms were summed to give a pattern glare score, with higher pattern glare scores indicating higher levels of visual stress.

**
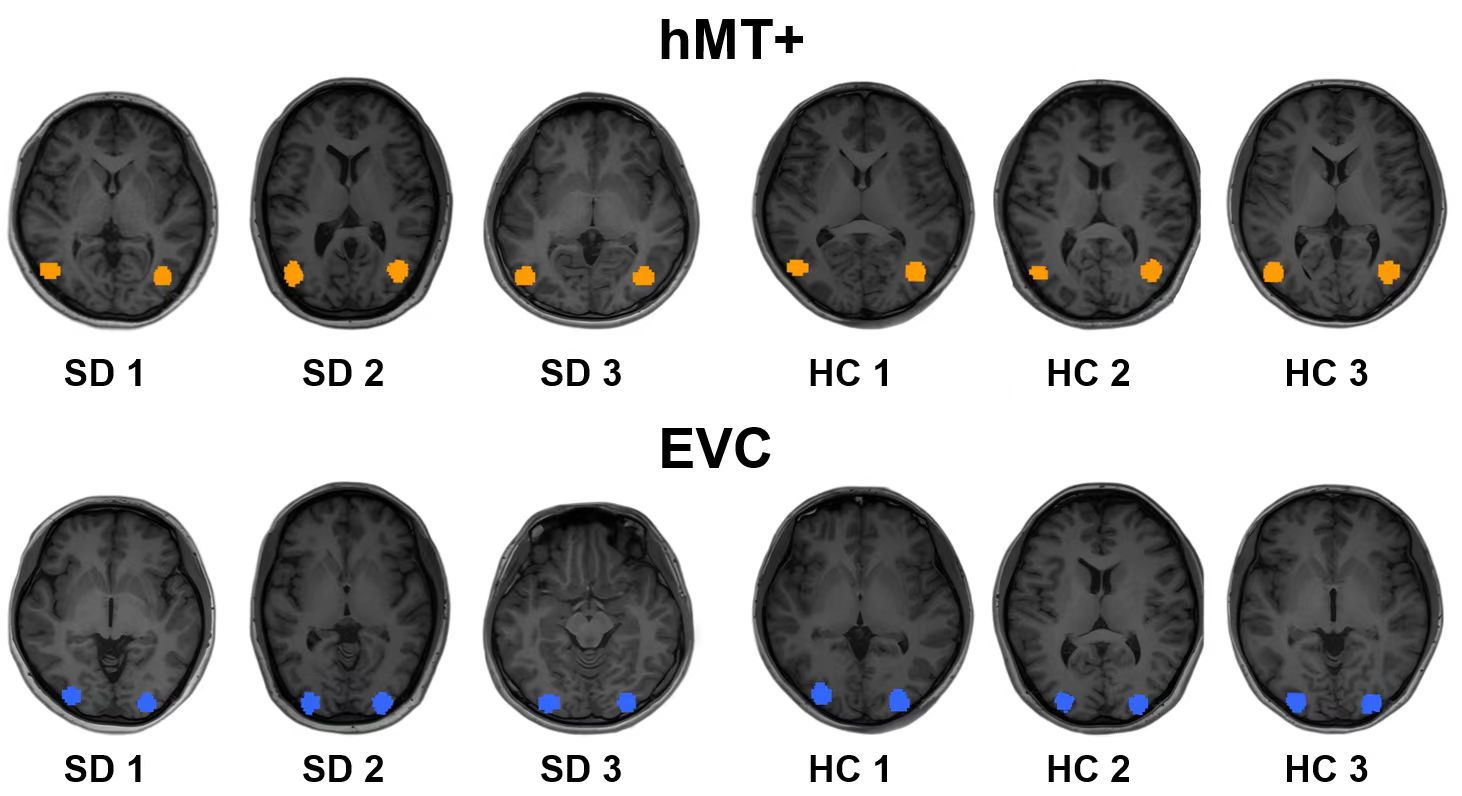
**

**Figure S1. Examples of the defined ROIs in hMT+ and EVC from six participants.** Abbreviations: hMT+, human middle temporal complex; EVC, early visual cortex; SD, subthreshold depression; HC, healthy control.

**Supplementary Results**


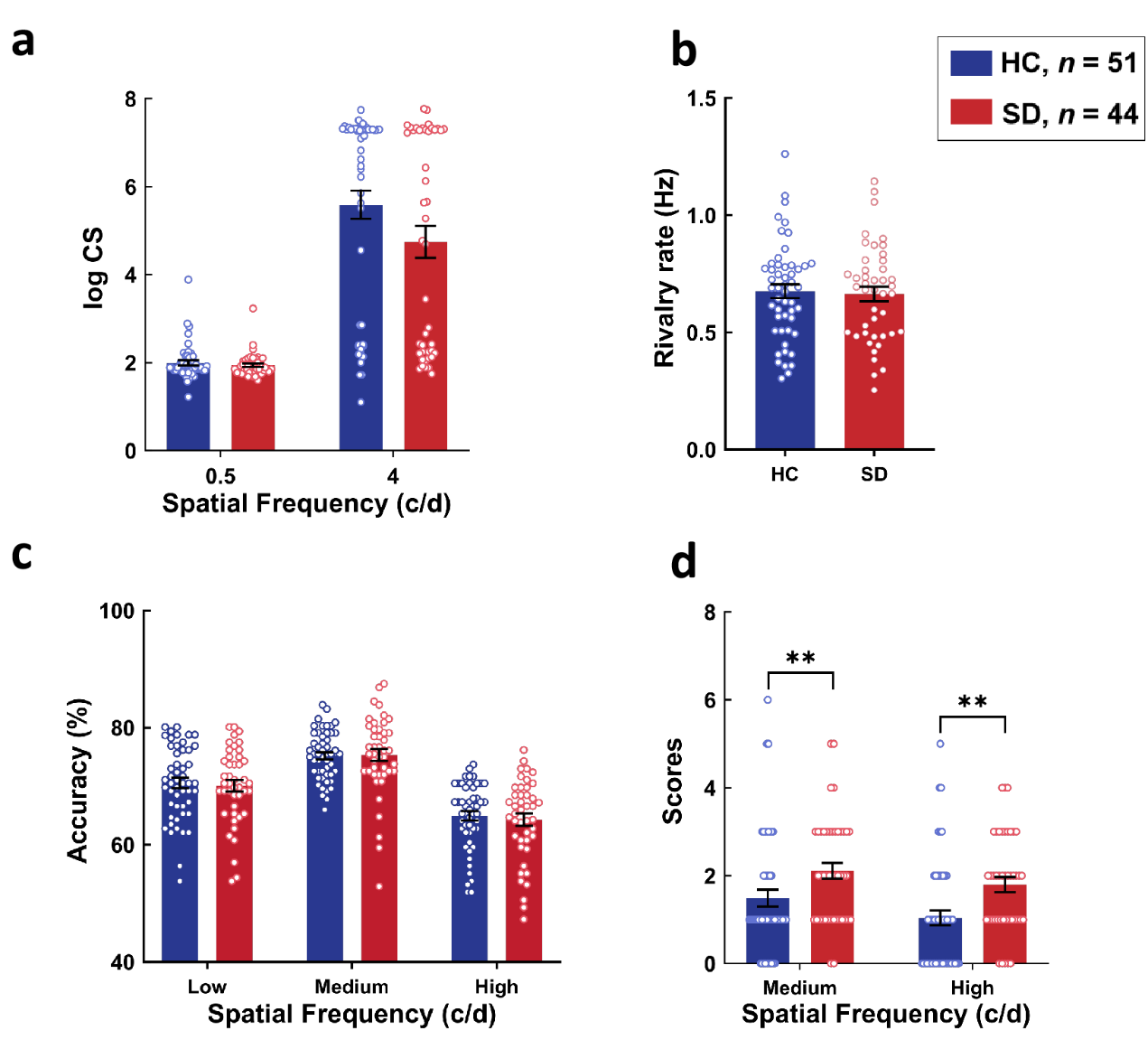


**Figure S2**. **Results of psychophysical behavioral experiments. a** Contrast sensitivity task: there was no significant main effect of group for log CS (*t*_184_ = 0.14, *p* = 0.889); **b** Binocular rivalry task: there was no significant between-group difference (*t*_93_ = 0.27, *p* = 0.790).; **c** Spatial frequency task: there was no significant main effect of group (*t*_195_ = -0.45, *p* = 0.653); **d** Pattern glare task: the pattern glare score was significantly higher in the SD group compared with the HC groups at both medium (z = -2.87, *p* = 0.004) and high spatial frequency gratings (z = -3.43, *p* < 0.001). Abbreviations: CS, contrast sensitivity; SD, subthreshold depression; HC, healthy control. ***p*< 0.01.

**REFERENCES**

1 Song XM, Hu XW, Li Z, Gao Y, Ju X, Liu DY *et al.* Reduction of higher-order occipital GABA and impaired visual perception in acute major depressive disorder. *Mol Psychiatry.* 2021; **26**: 6747–6755.

2 Chung STL, Legge GE. Comparing the Shape of Contrast Sensitivity Functions for Normal and Low Vision. *Invest Ophthalmol Vis Sci.* 2016; **57**: 198–207.

3 Zemon V, Herrera S, Gordon J, Revheim N, Silipo G, Butler PD. Contrast sensitivity deficits in schizophrenia: A psychophysical investigation. *Eur J Neurosci.* 2021; **53**: 1155–1170.

4 Blake R, Logothetis NK. Visual competition. *Nat Rev Neurosci* 2002; **3**: 13–21.

5 Patel A, Maurer D, Lewis TL. The development of spatial frequency discrimination. *J Vis.* 2010; **10**: 41.

6 O’donnell BF, Potts GF, Nestor PG, Stylianopoulos KC, Shenton ME, Mccarley RW. Spatial frequency discrimination in Schizophrenia. *J Abnorm Psychol*. 2002; **111**: 620–625.

7 Evans BJW, Stevenson SJ. The Pattern Glare Test: A review and determination of normative values. *Ophthalmic Physiol Opt.* 2008; **28**: 295–309.

8 Wilkins AJ, Evans BJW. Pattern glare test instructions. *IOO Sales Ltd, London* 2001.
