# Supplementary material for: Reduced neural suppression at occipital cortex in subthreshold depression
Source: Transl Psychiatry. 2025 Jul 1;15:220. doi: 10.1038/s41398-025-03446-9 (PMC12216281; doi:10.1038/s41398-025-03446-9)
Supplement: Supplementary file 2 — Table S1 [file 41398_2025_3446_MOESM2_ESM.docx]

**Table S1** Functional connectivity analysis with hMT+ as a seed region revealed enhanced functional connectivity in the SD group compared to the HC group. The significant threshold was set at a threshold of voxel-level *p* < 0.001 and a cluster-level family-wise error corrected threshold of *p* < 0.05. Abbreviations: hMT+, human middle temporal complex; HC, healthy control; SD, subthreshold depression; ACC, anterior cingulate cortex; mPFC, medial prefrontal cortex; PoCG, postcentral gyrus; L, left; R, right.

| Cluster | L/R | Cluster  Size  (#voxels) | *t* | MNI coordinates | | |
| --- | --- | --- | --- | --- | --- | --- |
|  |  |  |  | *x* | *y* | *z* |
| ACC | R | 158 | 5.75 | 14 | 22 | 28 |
| mPFC | R | 1,207 | 5.52 | 3 | 22 | 43 |
| PoCG | L | 1,788 | 5.44 | 26 | -30 | 60 |
| PoCG | R | 1,126 | 5.32 | -32 | -30 | 54 |
| insula | R | 35 | 4.75 | 32 | -28 | 20 |
